# An Unusual Presentation of Metastatic Breast Carcinoma as Cold Autoimmune Hemolytic Anemia

**DOI:** 10.4274/tjh.2014.0373

**Published:** 2015-02-15

**Authors:** Anand Chellappan, Chanaveerappa Bammigatti, Swaminathan Palamalai

**Affiliations:** 1 Jawaharlal Institute of Postgraduate Medical Education and Research (JIPMER), Department of Internal Medicine, Puducherry, India

**Keywords:** Cold-reactive antibody, Autoimmune hemolytic anemia, Breast carcinoma

## TO THE EDITOR

Cold autoimmune hemolytic anemia is a rare disease caused by cold-reactive antibodies. It has been reported in patients with lymphoproliferative disorders and autoimmune and infectious diseases, and rarely with solid tumors [1,2,3]. Herein we bring to your attention a patient with metastatic breast carcinoma who presented with cold autoimmune hemolytic anemia and showed a dramatic improvement following treatment with steroids.

**Figure 1:**
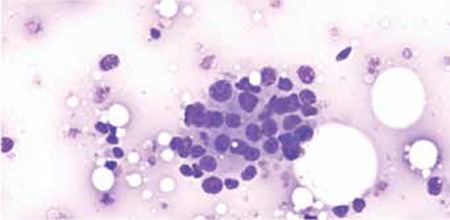
Bone marrow aspirate showing clusters of atypical epithelial cells consistent with metastatic adenocarcinoma deposits.

A 50-year-old woman presented with complaints of dyspnea, easy fatigability, and palpitations of 1 week in duration. Dyspnea had worsened rapidly and was present even at rest. She had been diagnosed with metastatic left breast carcinoma, stage T4b N1 M1, and had been treated with left mastectomy and chemotherapy. She was receiving second-line adjuvant chemotherapy with gemcitabine and capecitabine. She was also a diabetic on metformin and glibenclamide. There was no history of intake of any other drug. She had severe pallor and mild icterus at presentation. Her hemoglobin was 28 g/L and peripheral smear showed agglutinated RBCs, nucleated RBCs, and polychromatophils. Total leucocyte count was 9.2x109/L (neutrophils: 62%, lymphocytes: 30%, band forms: 3%, myelocytes: 4%, metamyelocytes: 1%) and platelet count was 165x109/L. Reticulocyte count was 7%. Direct antiglobulin test (DAT) was 3+ (graded on a scale from 0 indicating no agglutination to 4+ indicating solid agglutination). DAT was positive for C3d and negative for IgG. Liver function tests showed mild elevation of bilirubin (total bilirubin: 2.2 mg/dL and direct bilirubin: 0.7 mg/dL on day 1; total bilirubin: 4.2 mg/dL and direct bilirubin: 0.8 mg/dL on day 2). Urine hemoglobin was positive. Serum LDH level was 360 IU/L (reference range: 60-200 IU/L). Antinuclear antibody, HIV, and hepatitis B and C serologies were negative. Serum B12, folate, and ferritin levels were normal. Serum haptoglobin and cold agglutinin titers could not be measured due to the lack of this facility in our institute. Bone marrow aspirate revealed clusters of atypical epithelial cells along with few osteoclasts, consistent with metastatic adenocarcinoma deposits (Figure 1). She was given one packed cell transfusion at presentation and was started on prednisolone at 1 mg/kg body weight. She had a dramatic clinical improvement and did not require further transfusions. Her hemoglobin had improved to 70 g/L at 2 weeks.

Cold agglutinin disease is a form of autoimmune hemolytic anemia characterized by the presence of circulating antibodies, usually of the IgM type. These cold antibodies have little, if any, activity at body temperature but display increasing affinity for RBCs as the temperature decreases towards 0 °C. They require complement activation and fixation for hemolysis to occur. Cold autoimmune hemolytic anemia can be primary when there is no underlying systemic disorder. Secondary cold autoimmune hemolytic anemia is usually reported in patients with leukemias and lymphomas. However, it has also been described in cancers of the lung, colon, cervix, and breast [4,5]. In most of these cases the cancer had already metastasized at presentation. The pathogenetic mechanism is poorly understood [6]. Some of the proposed hypotheses include release of tumor-associated antigens mimicking RBC antigens, production of autoantibodies by the tumor itself, and adsorption of immune complexes on the erythrocyte membrane [6]. It is important to distinguish autoimmune hemolytic anemia from other non-immune causes of anemia like bone marrow infiltration by the tumor, since the treatment and prognosis vary accordingly. Our patient had evidence of bone marrow metastasis at presentation. Autoimmune hemolysis was confirmed by a positive direct antiglobulin test. Drug-induced hemolytic anemia is another important cause of autoimmune hemolysis. The most common drugs implicated are antimicrobials (e.g., cefotetan, ceftriaxone, and piperacillin). The only way to support a diagnosis of drug-induced hemolytic anemia is to see if a hematological remission occurs after withdrawal of the drug. Informed consent was obtained.

Autoimmune hemolytic anemia requires treatment in most cases. There are few studies addressing the treatment of cold autoimmune hemolytic anemia. This can include pharmacotherapy with corticosteroids, immunosuppressive drugs, and rituximab, among other agents [7]. Varying results have been obtained with the use of these agents. Steroid treatment is found to be much less effective in paraneoplastic autoimmune hemolytic anemia than in idiopathic autoimmune hemolytic anemia. Steroids are able to control hemolysis in only 15% of cases of cold hemagglutinin disease [8]. Treatment of the underlying malignancy and avoidance of cold exposure form important aspects of treatment. The improvement in hemoglobin in our case could be attributed to the treatment of the underlying breast malignancy and the use of steroids.

In conclusion, autoimmune hemolytic anemia is a potentially treatable cause of anemia in a patient with breast malignancy and the clinician should be aware of this complication.
